# Heightened aversion to risk and loss in depressed patients with a suicide attempt history

**DOI:** 10.1038/s41598-017-10541-5

**Published:** 2017-09-11

**Authors:** Kwangyeol Baek, JaeHyung Kwon, Jeong-Ho Chae, Yong An Chung, Jerald D. Kralik, Jung-Ah Min, HyuJung Huh, Kyung Mook Choi, Kuk-In Jang, Na-Bin Lee, Sunyoung Kim, Bradley S. Peterson, Jaeseung Jeong

**Affiliations:** 1 Department of Bio and Brain Engineering, Korea Advanced Institute of Science and Technology (KAIST), Daejeon, 34141 Republic of Korea; 2 Department of Psychiatry, University of Cambridge, Addenbrooke’s Hospital, Cambridge, CB2 0QQ United Kingdom; 3 Department of Psychiatry, College of Medicine, The Catholic University of Korea, Seoul, 06591 Republic of Korea; 4 Department of Radiology, College of Medicine, The Catholic University of Korea, Seoul, 06591 Republic of Korea; 5 Institute for the Developing Mind, Children’s Hospital Los Angeles and the Keck School of Medicine at the University of Southern California, Los Angeles, CA 90033 United States

## Abstract

Suicide attempters have been found to be impaired in decision-making; however, their specific biases in evaluating uncertain outcomes remain unclear. Here we tested the hypothesis that suicidal behavior is associated with heightened aversion to risk and loss, which might produce negative predictions about uncertain future events. Forty-five depressed patients with a suicide attempt history, 47 nonsuicidal depressed patients, and 75 healthy controls participated in monetary decision-making tasks assessing risk and loss aversion. Suicide attempters compared with the other groups exhibited greater aversion to both risk and loss during gambles involving potential loss. Risk and loss aversion correlated with each other in the depressed patients, suggesting that a common pathophysiological mechanism underlies these biases. In addition, emotion regulation via suppression, a detrimental emotional control strategy, was positively correlated with loss aversion in the depressed patients, also implicating impairment in regulatory processes. A preliminary fMRI study also found disrupted neural responses to potential gains and losses in the subgenual anterior cingulate cortex, insula cortex, and left amygdala, brain regions involved in valuation, emotion reactivity, and emotion regulation. The findings thus implicate heightened negative valuation in decision-making under risk, and impaired emotion regulation in depressed patients with a history of suicide attempts.

## Introduction

Suicide is an extreme example of irrational decision-making and is most prevalent in depressed patients. Depression appears to involve a biased processing of negative information and a reduced sensitivity to reward, which together can distort an individual’s valuation of prospective outcomes^1^. However, how these biases in information processing in depressed patients specifically affect their decision-making and their risk of attempting suicide remains unclear.

Increasing evidence suggests the presence of impaired decision-making under conditions of uncertainty in depressed patients, particularly in individuals who have attempted suicide. Conventional neuropsychological tasks have found that executive functioning is impaired in depressed patients with prior suicide attempts^2^ and in those with current suicide ideation^3^. Patients with affective disorders are also impaired in decision-making on the Iowa Gambling Task (IGT)^4–7^. A history of attempting suicide in affective patients was specifically associated with poor learning of long-term advantageous choices in the IGT^5, 6, 8^. Depressed patients were also impaired in performance on a reversal learning task,^9, 10^ which, similar to the IGT, requires adaptive learning from positive and negative feedback. Among elderly depressed patients, suicide attempters also exhibited impaired reversal learning^11^ and a blunted response in the subgenual part of the anterior cingulate cortex^12^, suggesting the presence of impaired reward learning.

Such IGT or reversal learning tasks in the previous studies assess adaptive learning in environments with epistemic uncertainty, i.e., incomplete knowledge about the structure of the environment and/or the distribution of outcomes. These types of tasks can capture complex decision-making ability in dynamic environments with feedback in serial choices, but they involve multiple cognitive and affective processes in decision-making: e.g., sensitivity to monetary reward and punishment, learning from feedback, executive function, etc.^13, 14^. Thus, it is difficult to characterize specific decision biases in depressed patients or suicide attempters using such complex learning tasks. Experimental tasks and models from neuroeconomics, the neuroscience of decision-making, help to extend this work by providing tools for investigating the valuation process in humans that are less confounded with learning and executive functioning. *Decision-making under risk* in neuroeconomics is defined as choosing among prospective outcomes, typically with explicit knowledge of value and probability. It does not generally involve learning from feedback, because it presents explicit information about the values and probabilities of the prospective outcomes. Decision-making under risk may thus capture potential valuation biases in aleatory uncertainty (uncertainty with explicit knowledge of outcome probability), a distinct type of uncertainty in the adaptive learning environment.

Two important phenomena related to valuation — risk and loss aversion — are commonly observed biases in decision-making under risk. *Risk aversion* is the preference for certainty over risk, even when the expected outcomes, averaged over time and repeated choices, are identical (e.g., preferring the certain gain of $50 over a gamble with a binary outcome that would yield, with a 50/50 probability, either $100 or nothing). *Loss aversion* denotes the weighting of potential losses substantially more than potential gains. Although both of these biases are found in the general population^15^, they are also particularly prevalent in depressed patients^16, 17^. By extension, a reasonable hypothesis is that these aversions would be even stronger in patients with a suicide attempt history, because the assessment of possible future outcomes might be especially negatively biased in this subset of depressed patients. For example, a recent neuroeconomics hypothesis of suicide^18^ postulated that strong risk and loss aversion can increase the likelihood of committing suicide because suicide is associated with one’s expectation of income uncertainty and cost of living. Heightened risk and loss aversion may amplify negative predictions about uncertain future events in general, and make one evaluate their life as less worth living. However, whether these aversions are actually heightened in depressed suicide attempters compared to other depressed patients, and the specific conditions under which the aversions may manifest, is unknown.

We aimed to test the hypothesis that aversion to risk and loss are heightened in depressed patients with a suicide attempt history compared to depressed patients without a prior attempt and compared to non-depressed healthy controls. We also assessed whether the aversion to risk in these patients manifests specifically in the loss domain, as for example when choosing between a certain loss and a gamble that could lead to either a greater loss or no loss at all. Finally, we conducted a preliminary fMRI experiment to investigate the neural substrates of this potentially distorted valuation in patients with a history of attempting suicide. We hypothesized that distorted valuation would be associated with neural activity in brain regions subserving emotion and the regulation of emotion.

## Results

### Behavior experiment

**Figure 1:**
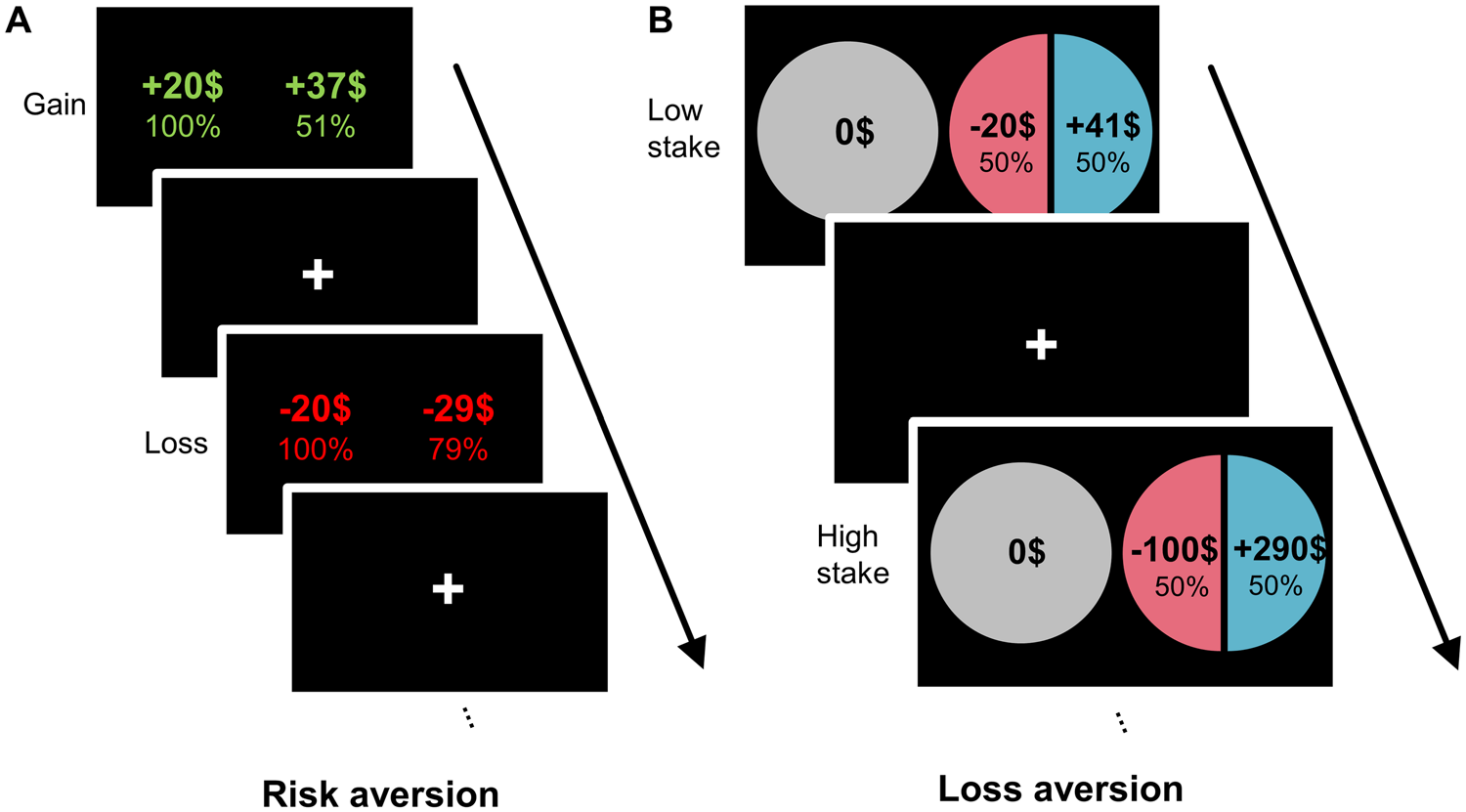
Monetary decision-making tasks to assess risk and loss aversion in valuation. (**A**) Risk aversion task. In the gain condition, participants chose between a certain monetary gain ($20) and a probabilistic gain of larger magnitude. In the loss condition, they chose between a certain monetary loss (-$20) and a probabilistic loss of larger magnitude (or no loss). The amount of the probabilistic gain (or loss) was adjusted dynamically depending on each participant’s choices in two consecutive trials with the same condition and probability, until the participant’s subjective value of the probabilistic gain (or loss) was equal to the certain gain (or loss) of $20. For example, when the participant chose a probabilistic gain (gamble choice) over a certain gain of $20 for the given probability twice in a row, the magnitude of the probabilistic gain was reduced in a stepwise manner. The discount rate of probabilistic gain (or loss) with respect to the odds against winning (or losing) was then calculated, and its logarithm value was obtained as a normalized measure of risk aversion for each individual (see Methods). (**B**) Loss aversion task. In every trial, participants chose between a 50-50 gamble or status quo ($0). The amount of gain in the 50-50 gamble was systematically adjusted in a similar manner as the risk aversion task in (**A**), so that the participant’s subjective value of the 50-50 gamble approached $0, the status quo. At this indifference point, the participant’s subjective value of potential gain in the 50-50 gamble was exactly negated by the potential loss in the same gamble; thus, loss aversion *λ* was calculated as the ratio of the amount of gain to the amount of loss. In lay terms, this task determines for each participant the amount of likely gain that is required to overcome the potential loss, if they are to elect to enter into a gamble.

**Figure 2:**
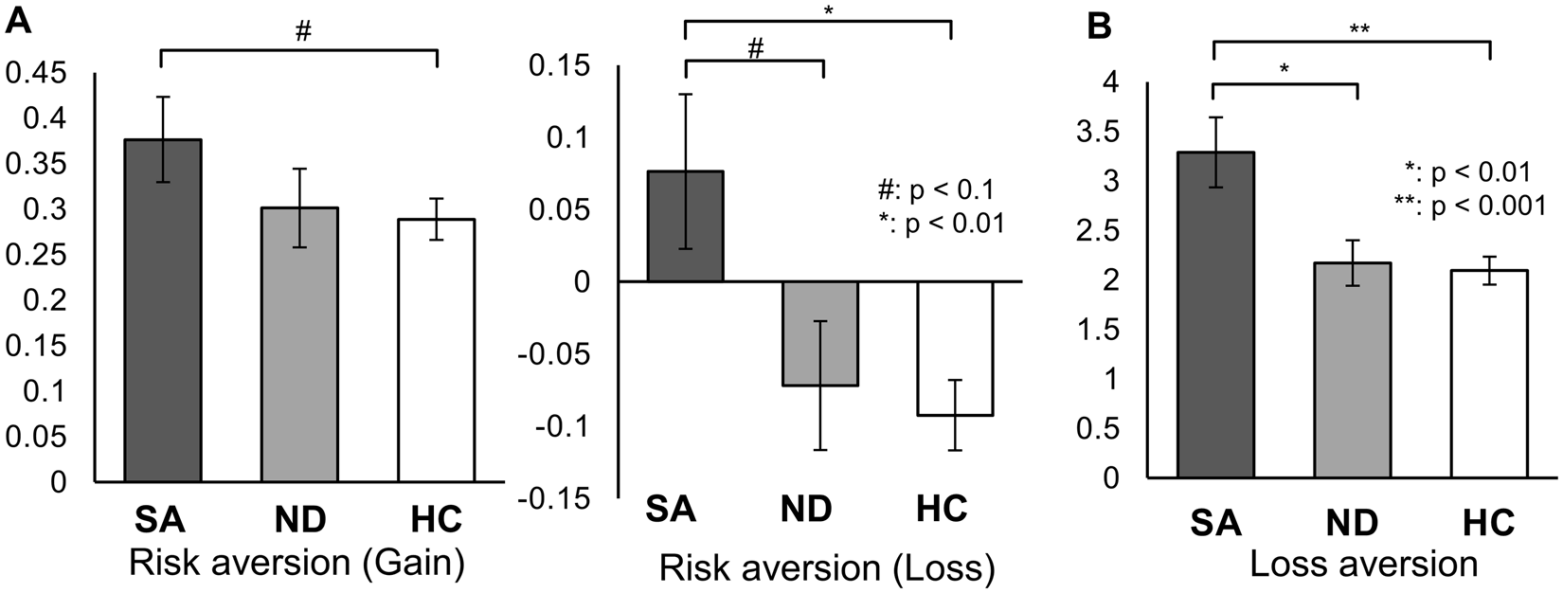
Increased risk aversion with probabilistic loss outcomes (−log *k*_*loss*_) and increased loss aversion (*λ*) in depressed patients with a suicide attempt history. SA: Suicide attempters, ND: Nonsuicidal depressed patients, HC: Healthy controls. (**A**) Suicide attempters showed increased risk aversion (left and middle graphs) ﻿﻿that reached significance in the loss condition (middle graph) compared to healthy controls. Suicide attempters generally exhibited risk aversion in the loss condition (−log *k*_*loss*_ > 0), whereas nonsuicidal patients and healthy controls preferred risk-taking for probabilistic loss outcomes (middle graph). (**B**) Suicide attempters exhibited significantly higher loss aversion than did nonsuicidal depressed patients or healthy controls.

We conducted monetary decision-making tasks to examine risk and loss aversion quantitatively in 45 depressed patients who previously attempted suicide, 47 depressed patients without a prior history of attempting suicide, and 129 healthy controls (Fig. 1). Demographic information about the participants is shown in Table 1. One-way ANOVA revealed a significant group difference in risk aversion in the loss condition—i.e., when participants were deciding whether to select either a certain loss of a specified amount (e.g., $20) or a gamble that would yield either (a) no loss or (b) a loss that was even greater than the sure loss (e.g., $29) (see the loss condition example in Fig. 1a). In contrast, we found no significant group difference in the gain condition, in which participants chose between a sure gain or a gamble for a potentially larger one. Indeed, the people with a previous suicide attempt history exhibited a higher risk aversion for loss (quantified as −log *k*_*loss*_) compared to both the depressed patients without a previous suicide attempt and healthy controls, as shown in Fig. 2A (middle graph, post hoc Tukey’s test: p = 0.06 and p < 0.01, respectively). In fact, whereas both the healthy controls and nonsuicidal depressed patients on average preferred the risk-taking option in the loss condition as predicted by Prospect theory^15^, the suicide attempters on average exhibited risk aversion, choosing the certain loss over a gamble that could yield either no loss or an even greater one.

**Table 1:** Demographic and clinical information of healthy controls and depressed patients. BIS/BAS: Behavioral Inhibition System/Behavioral Approach System score, ERQ: Emotion Regulation Questionnaire.

	Healthy Controls (n = 75)	Nonsuicidal Depressed Patients (n = 47)	Suicide Attempters (n = 45)	P *value*
Male sex: number (%)	46 (61)	22 (47)	24 (53)	0.28^a^
Age: mean (SD), years	25.4 (4.6)	26.8 (6.3)	24.5 (5.9)	0.10
Education: mean (SD), years	13.6 (1.9)	14.0 (2.2)	13.1 (1.9)	0.11
Antidepressant treatment: number (%)	N/A	25 (53)	25 (56)	0.82^a^
Current suicidal ideation: number (%)	N/A	18 (38)	31 (66)	0.003^a^
Questionnaires: mean (SD)				
Beck Depression Inventory	4.4 (3.0)	25.3 (7.8)	35.4 (10.7)	<0.001^b^
Beck Hopelessness Scale	9.3 (1.0)	43.9 (5.6)	47.9 (6.4)	<0.001^b^
Suicide Ideation Scale	1.8 (2.5)	13.7 (15.5)	20.6 (5.2)	<0.001^b^
State Anxiety Inventory	32.6 (4.7)	58.6 (11.3)	65.8 (9.4)	<0.001^b^
Trait Anxiety Inventory	33.6 (5.1)	61.7 (9.0)	66.4 (8.4)	<0.001^b^
Barrat Impulsivity Scale	46.6 (7.0)	52.9 (7.1)	54.9 (10.3)	<0.001^c^
BIS/BAS – Behavioral inhibition	16.7 (2.9)	22.4 (4.1)	23.6 (3.3)	<0.001^c^
BIS/BAS – Reward responsivity	15.7 (3.3)	15.1 (2.6)	15.3 (3.6)	0.71
BIS/BAS – Drive	11.8 (1.9)	9.3 (2.2)	10.3 (3.2)	<0.001^d^
BIS/BAS – Fun seeking	11.4 (1.9)	9.7 (2.5)	10.0 (3.3)	0.011^e^
ERQ – Reappraisal	N/A	22.0 (5.8)	20.4 (4.6)	0.57
ERQ – Suppression	N/A	14.0 (6.1)	17.8 (6.0)	0.02^f^

^a^χ^2^ test.

^b–e^One-way ANOVA, p < 0.05 in post-hoc Tukey’s test.

^b^Suicide attempters > Nonsuicidal depressed patients > Healthy controls.

^c^Suicide attempters > Healthy controls; also Nonsuicidal depressed patients > Healthy controls.

^d^Healthy controls > Suicide attempters; also Healthy controls > Nonsuicidal depressed patients.

^e^Healthy controls > Nonsuicidal depressed patients.

^f^Two-sample *t* test.

Loss aversion was also significantly increased in the prior suicide attempters compared to both the nonsuicidal depressed patients and healthy controls (p < 0.01 and p < 0.001, respectively. Figure 2B). The latter two groups did not differ significantly from one another in either risk or loss aversion.

**Figure 3:**
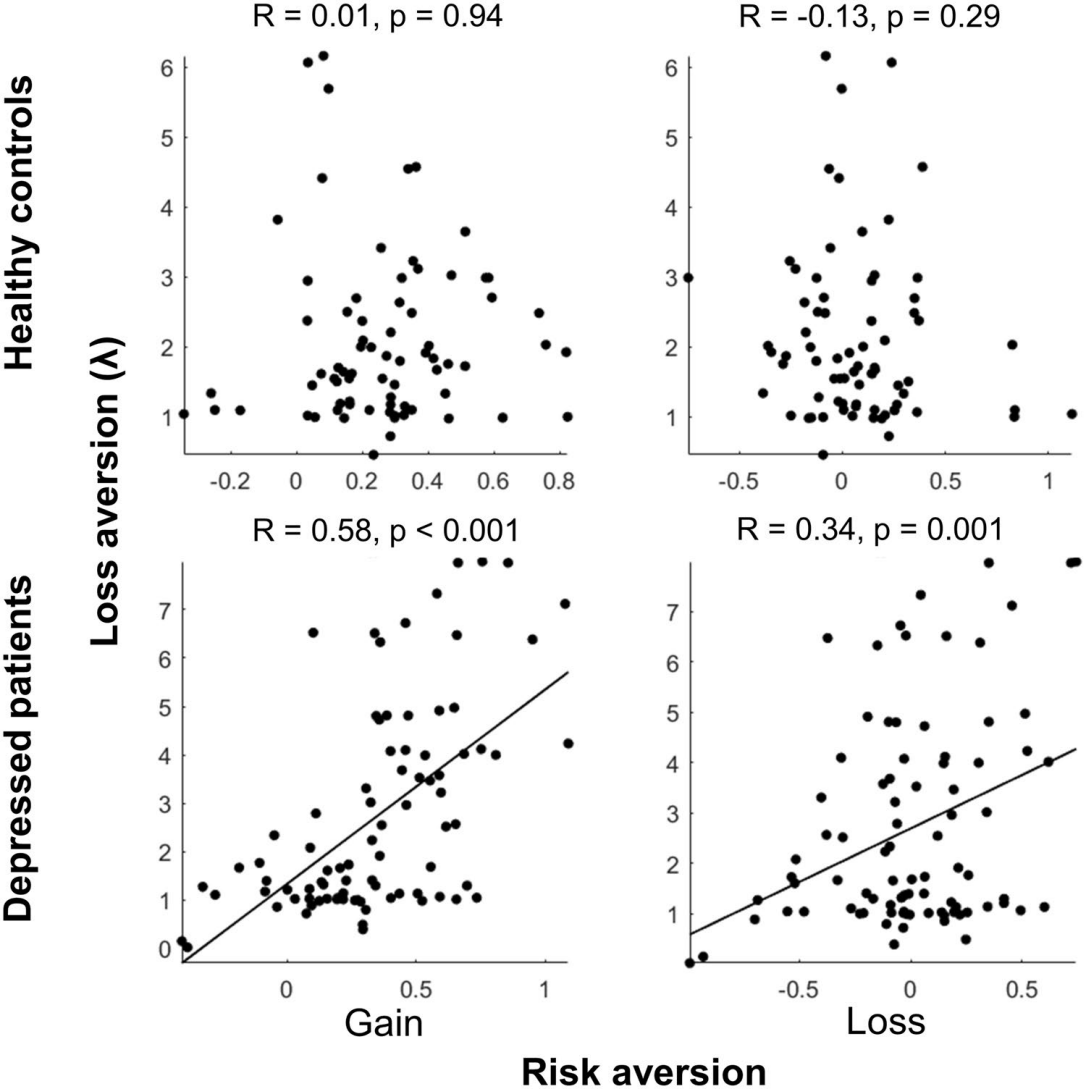
Correlations between risk aversion and loss aversion. Significant correlations were observed only in depressed patients.

In addition, we found that risk and loss aversion were not significantly inter-correlated in healthy controls in either the gain or loss conditions, but they were significantly positively correlated in all depressed patients regardless of prior suicidal attempts, in both the gain and loss conditions (R = 0.58, p < 0.001 for gain; R = 0.34, p < 0.01 for loss; Fig. 3). The correlations observed in the depressed patient group were significantly higher than the correlations in the healthy controls (Fisher’s z-transformation: Z = 4.14, p < 0.001 for gain; Z = 3.04, p < 0.01 for loss.). This inter-correlation of risk and loss aversion in depressed patients suggests a common underlying pathogenesis for altered risk and loss aversion in this patient population that may become especially exacerbated in the suicide-attempters.

In addition to the risk and loss aversion tasks, the clinical patients were interviewed and all three groups filled out a series of questionnaires (Table 1). Regarding depression in general, the questionnaires revealed a higher sensitivity to aversive conditions (i.e., higher behavioral inhibition), reduced impulse control and lower drive in both patient populations compared to the control participants. Then, with respect to those who also attempted suicide, the questionnaires revealed that, compared to the other two groups, the suicide attempters scored higher on symptoms of depression, hopelessness, suicidal ideation, and both state and trait anxiety. They also scored higher than the depressed patients without a prior suicide attempt on the use of a suppression strategy when regulating emotions. The interviews also revealed a higher rate of current suicidal ideation in the suicide attempters group compared to the nonsuicidal depressed patients. In contrast, we found no difference between the groups for reward responsivity, corroborating the results in the gain condition of the risk aversion task, in which we found no differences between groups.

To begin to examine how closely the risk and loss aversion findings relate to the other factors underlying suicide attempts revealed by the interviews and questionnaires, we first examined the correlations between the questionnaire scores and the behavioral task measures for (a) all three groups together (when possible, i.e., all except for the ERQ) and (b) the depressed patients only (i.e., suicide and nonsuicidal attempters together). We found that the suppression score correlated positively with loss aversion in depressed patients (R = 0.35, p = 0.017). No other questionnaire scores correlated with risk or loss aversion for either all groups together or in the depressed patients alone. We also found no effect of current suicide ideation or current antidepressant treatment on risk or loss aversion in depressed patients. This lack of effect for either suicide ideation or antidepressant treatment in the depressed patients was verified with two-sample t-tests (Supplementary Table 1) and three-way ANOVAs that revealed a significant effect of suicide attempt history (p = 0.019 for risk aversion for loss, and p = 0.009 for loss aversion) but not for current suicide ideation or antidepressant medication (p > 0.127).

### Preliminary fMRI experiment

**Figure 4:**
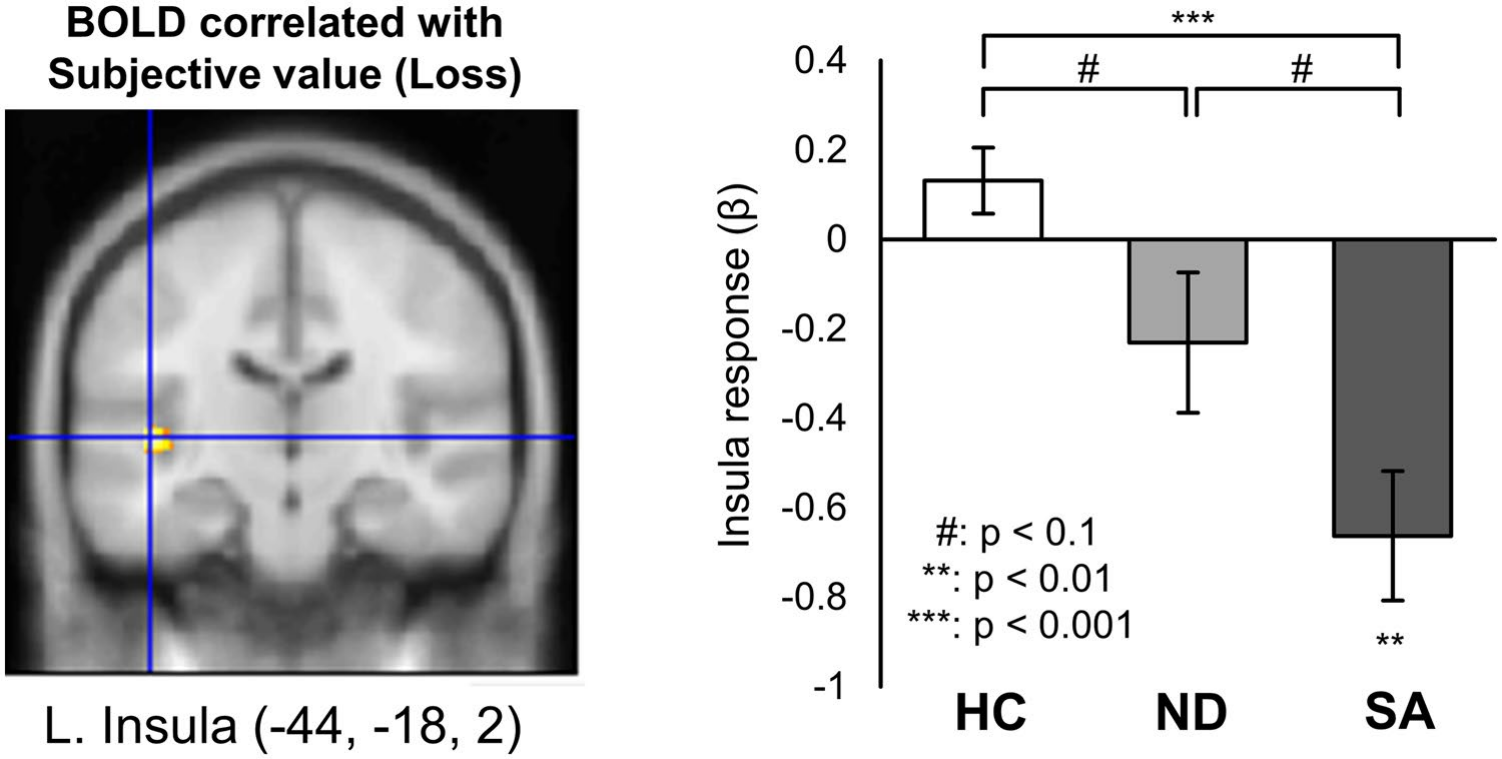
Group difference in insular activity correlated with subjective value of probabilistic loss in the risk aversion task. SA: Suicide attempters, ND: Nonsuicidal depressed patients, HC: Healthy controls. A one-way ANOVA across groups (p < 0.05, small volume corrected) revealed a significant overall group difference, and in particular, a significant difference between the suicide attempters and healthy controls (p < 0.001, post hoc Tukey’s test). Left insula activity was significantly correlated with subjective loss in the suicide attempters only, such that activity decreased with size of loss (p < 0.01, one-sample *t* test). Mean ± S.E.M.

To begin to identify the neural correlates of risk and loss aversion in suicide attempters, we conducted a preliminary fMRI experiment on 22 patients diagnosed with depression (10 suicide attempters) and 22 healthy controls. As shown in Fig. 4, in the loss condition of the risk aversion task, we found a significant group difference in the degree to which the neural activity of the left insula correlated with the subjective values of probabilistic loss for each probability profile (one-way ANOVA: p < 0.05, small volume corrected). In particular, there was a significant difference between the suicide attempters and healthy controls (post hoc Tukey’s test: p < 0.001). The insula activity in the nonsuicidal depressed patients was at an intermediate level between the suicide attempters and healthy controls, with a marginal significance level when directly compared to each group (p < 0.1, post hoc Tukey’s test). Examining the average correlations between neural activity and subjective loss (the beta weights) individually for each group, neural activity of the left insula negatively covaried with subjective loss (i.e., lower activity with higher losses) in the suicide attempters (p < 0.01, one-sample t-test), but not in the other two groups. In an exploratory whole-brain analysis within each group (p < 0.001, uncorrected), insula activity correlated significantly (and again negatively) with the subjective value of probabilistic gain and loss only in the suicide attempters (Supplementary Tables S2 and S3).

**Figure 5:**
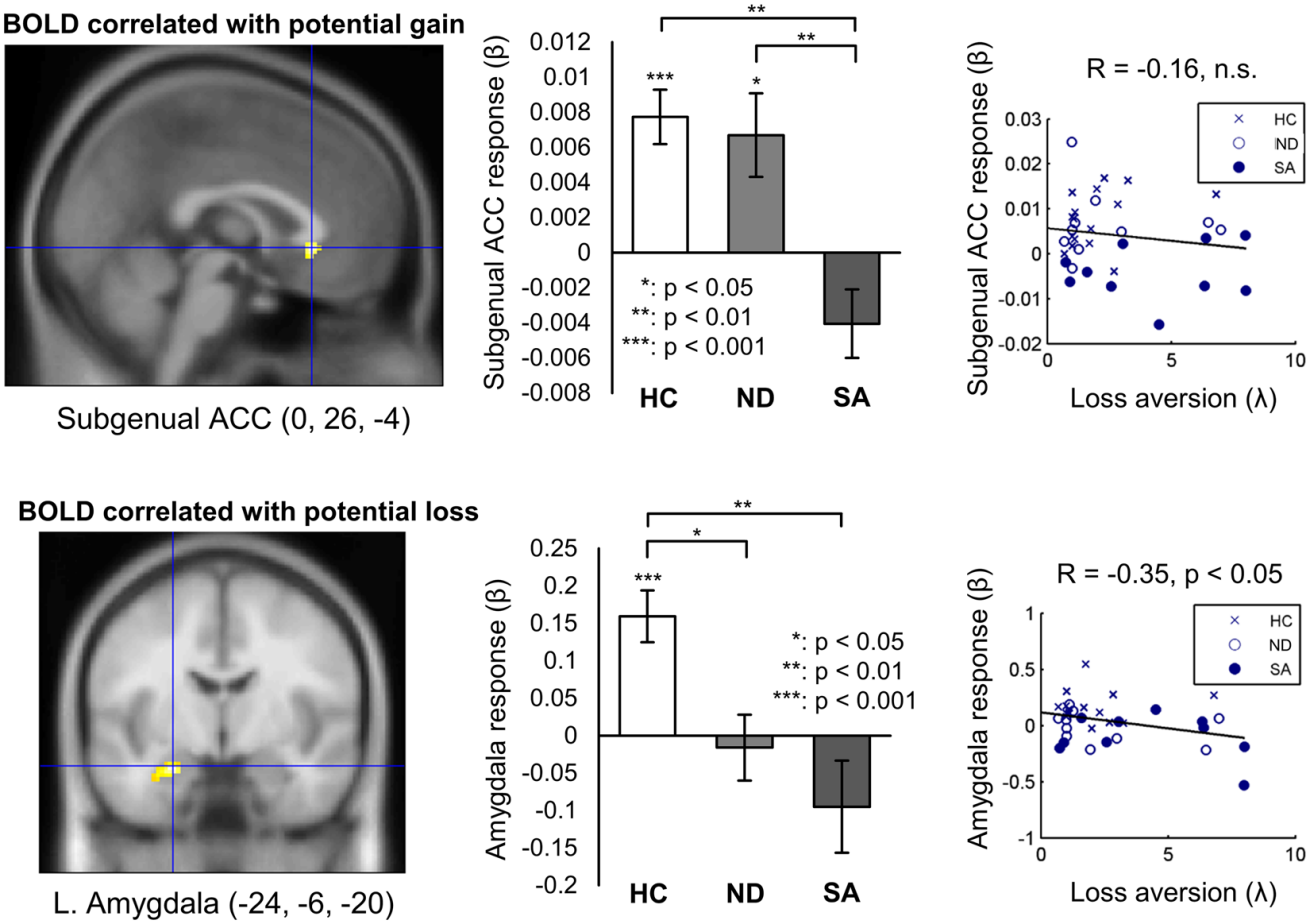
Group difference in BOLD response that covaried with the amount of potential gain (subgenual ACC) and loss (amygdala) in 50-50 gambles of the loss aversion task. SA: Suicide attempters, ND: Nonsuicidal depressed patients, HC: Healthy controls. **Top panel**. There was an overall group difference in the relationship of sgACC activity with the amount of potential gain in the task (one-way ANOVA across all groups, p < 0.05, small volume corrected); the suicide attempters showed a blunted or reversed pattern of sgACC activity compared to both the healthy controls and nonsuicidal depressed patients (p < 0.01 for both comparisons, post hoc Tukey’s test). Both the healthy controls and nonsuicidal depressed patients exhibited significant positive correlations in sgACC activity with the amount of potential gain in the task (p < 0.001 and p < 0.05, respectively, one-sample t-test). Mean ± S.E.M. **Bottom panel**. There was a significant overall group difference in the relationship of left amygdala activity to potential loss (one-way ANOVA: p < 0.05, small volume corrected), with both depressed groups exhibiting blunted or reversed left amygdala reactivity to the amount of potential loss when compared to healthy controls (p < 0.01 and p < 0.05, respectively, post hoc Tukey’s test). The positive correlation of potential loss with left amygdala activity in the healthy controls was significant (p < 0.001, one-sample t-test). Among all participants, the individual variation in the amygdala response correlated significantly with loss aversion estimated in the behavioral experiment (right bottom panel). Mean ± S.E.M.

In the loss aversion task, we found a significant group difference in the relationship of neural activity in the subgenual anterior cingulate cortex (sgACC) to the amount of potential gain in the 50-50 gamble (one-way ANOVA: p < 0.05, small volume corrected), with the prior suicide attempters exhibiting a blunted or reversed neural response to potential gain in the sgACC compared with the nonsuicidal depressed patients and healthy controls (p < 0.01 for both comparisons, post hoc Tukey’s test; Fig. 5). Examining the average correlations (beta weights) between neural activity in the sgACC and potential gain individually for each group, both the healthy controls and nonsuicidal depressed patients exhibited significant positive correlations (Fig. 5; p < 0.001 and p < 0.05, respectively, one-sample t-test).

In the loss aversion task, we also found a significant overall group difference in the relationship of left amygdala activity to potential loss (one-way ANOVA: p < 0.05, small volume corrected), with the neural response in the left amygdala in both the suicide attempters and nonsuicidal depressed patients being blunted compared to the healthy controls (p < 0.01 and p < 0.05, respectively, post hoc Tukey’s test). Examining the average correlations between neural activity and subjective loss (the beta weights) individually for each group, as with sgACC and potential gain, the healthy controls exhibited a significant positive correlation between left amygdala activity and the amount of potential loss (p < 0.001, one-sample t-test). The left amygdala response to potential losses also correlated with the level of loss aversion across all subjects (R = −0.35, p < 0.05), which indicates that amygdala reactivity was related to the observed behavioral loss aversion.

No other group difference was found in a whole-brain analysis (one-way ANOVA) at a significance level of an uncorrected p < 0.001 and an extent threshold k > 10. Significant BOLD activations observed within each group in this whole-brain exploratory analysis are listed in Supplementary Tables S2~S5.

## Discussion

### Risk aversion to potential losses and heightened loss aversion in suicidal patients

In the risk aversion task, previous suicide attempters were, on average, risk-averse during the loss condition, preferring a certain outcome over a gamble when faced with potential losses from either choice. Both the healthy controls and depressed patients without a prior suicide attempt, in contrast, preferred on average to gamble rather than to accept a loss that was certain to happen. The healthy control group’s bias toward risk-taking in the loss domain could represent either (a) a desire to avoid any losses or (b) some insensitivity to the difference in the certain and probabilistic loss amounts (i.e., why not gamble if not that much more of a loss). In contrast, the heightened risk aversion in the suicide attempters does not appear to stem from a stronger desire to avoid any losses, since that presumably would serve to increase risk-taking rather than reduce it, suggesting instead that the heightened risk aversion may derive from a greater sensitivity to the difference in the amounts of loss in the certain and probabilistic choices. The greater sensitivity to loss is further supported by the increased loss aversion in the suicide-attempt-history group, in which the units of loss are much greater than the units of gain. Taken together, then, our results suggest that suicide attempters have a heightened sensitivity to loss, which in turn implicates the valuation process in the pathogenesis of suicide attempts. We also cannot rule out an additional possible interaction, such that the patients who attempted suicide may overestimate probabilities in the context of losses (with the likelihood of losing even more money appearing higher).

Thus, patients with heightened loss aversion might estimate potential negative events (e.g., disemployment, social rejection, disease, etc.) in the future to be much more negatively valued, and thus may evaluate their life as less worth living. Patients with increased risk aversion in loss may also overestimate the likelihood of undesirable events in the future. In other words, suicide can be the most extreme choice of avoiding what is anticipated to be a more highly aversive future.

We also found significant positive correlations between loss and risk aversion in both the gain and loss domains for the depressed patients, correlations that were not significant in the healthy controls. The covaried aversions to risk and loss in depressed patients suggests that a common psychopathological basis might underlie the valuation biases, such as an increased focus on the worse than expected outcomes in all three conditions^9, 10, 19^, but future examination is required to isolate the specific task variables and underlying psychological and neural mechanisms. Nonetheless, the results suggest that this correlation between risk and loss aversion could be another possible behavioral phenotype of depressive disorders, in general, and could be used to identify depressed patients potentially at risk for attempted suicide, more specifically.

In addition to our behavioral decision-making paradigm, questionnaires and interviews also enabled further investigation of other factors potentially related to depression and suicide behavior, such as the level of subjective well-being, perceived stress and explicit expectations of future life events. With respect to depression in general, both patient populations revealed a higher sensitivity to aversive conditions (i.e., heightened behavioral inhibition), reduced impulse control and lower drive compared to the control participants. For the depressed patients who actually attempted suicide, they scored higher on symptoms of depression, hopelessness, suicidal ideation, and both state and trait anxiety than both the nonsuicidal depressed patient and control groups, and higher than the nonsuicidal depressed patients on the use of a suppression strategy when regulating emotions. Thus, as expected, multiple factors were found to underlie suicidal behavior. Then, to begin to investigate the potential links between these additional factors with both risk and loss aversion, we examined their correlations. However, we only found a significant (positive) relationship between the suppression score and loss aversion in the depressed patients. Moreover, we found no evidence for a significant relationship of either current suicide ideation or current antidepressant treatment with either risk or loss aversion. Thus, no other examined factors besides suppression were found to relate to risk or loss aversion. This apparent lack of a stronger linear relationship of risk or loss aversion with these other important factors may be due to methodology (e.g., inherent lack of precision in questionnaire assays) or an actual weaker relationship among the variables. A weaker relationship may suggest that risk and loss aversion affect suicidal thoughts and behavior more independently from the other factors, potentially reflecting a separable dysfunctional process such as valuation. In any case, the lack of a stronger linear relationship with other known influential factors provides additional evidence that loss aversion and risk aversion with losses appear to be novel and separable *indicators* of potential suicide risk. Moreover, the rigorous and quantitative nature of the neuroeconomics paradigm should help to elucidate the detailed nature of the interrelationships in future studies.

Given that the suicide attempters (a) had a higher score for the suppression strategy in emotion regulation compared to the nonsuicidal depressed patients, and (b) a score that also correlated with loss aversion in the depressed patients, it suggests that the heightened loss aversion (or negative valuations) is associated with impaired regulation of negative affect in the depressed patients^20, 21^. Several previous studies have suggested a role of emotion regulation in aversion to risk and loss. Sokol-Hessener *et al*.^22, 23^ reported that intentional emotion regulation strategies (e.g., reappraisal-focused) reduced loss aversion and related physiological responses during a monetary decision-making task. In another study using the Columbia Card Task, a cognitive reappraisal strategy was associated with increased risk-taking and a suppression strategy was associated with decreased risk-taking^24^. Here, we found that the emotion regulation strategy of suppression associated positively with loss aversion, such that increased use of the strategy was accompanied by heightened loss aversion. As the use of the suppression strategy is typically known to be detrimental compared to the cognitive reappraisal strategy, it suggests that either the heightened sensitivity to loss promotes the use of suppression or that the strategy itself leads to the heightened sensitivity. Determining exactly how these factors are causally related, therefore, warrants further study. Further research is also necessary to examine the potential interrelationships with other conceptually related factors, such as those of the interpersonal-psychological theory of suicide behavior related to emotion dysregulation: e.g., low distress tolerance and high negative urgency associated with perceived burdensomeness and thwarted belongingness in recent studies^25, 26^.

Finally, the observed valuation biases can also facilitate an understanding of the decision-making deficits observed in previous studies that used adaptive learning tasks. An over-reactivity to negative feedback has typically been observed in depressed patients^9, 10, 19^, and it could be affected by excessive loss aversion in the valuation process. In contrast, heightened aversion to risk and loss appears to contrast with previous findings of impaired performance on the IGT in depressed or suicidal patients^5, 27^, given that disadvantageous decks that they *preferred* in the IGT have a *larger* loss and more variance in outcomes. However, the IGT does not depend solely on valuation processes but also requires other cognitive functions, such as adaptive learning. In addition, other aspects of the IGT prove challenging to interpret even in terms of valuation: for example, in the framework of Prospect theory a smaller gain in advantageous decks can be considered a loss compared with the larger gain in disadvantageous decks^15^. To disentangle valuation effects from other related factors like feedback learning, cognitive neuroeconomic task paradigms such as what we utilized here, as well as elaborated computational models (e.g., a reinforcement learning model with valence parameters), should prove valuable in future studies^5^.

### Altered brain circuitry of valuation and emotion regulation

We found group differences in the BOLD activity of paralimbic brain regions, such as the insula, sgACC, and amygdala, which are known to be related to valuation, emotion reactivity, and emotion regulation. Insula activity in prior suicide attempters negatively correlated significantly with the subjective value of probabilistic loss, such that activity decreased with the degree of loss, a finding that was not observed in the other two groups. The insula activity would thus appear to be related to their risk-aversive tendency and their heightened negative valuation of expected loss. The intermediate value for nonsuicidal depressed patients also suggests that the insular activity may be related to the intermediate level of their discount rate in the loss condition, −log(K_loss_), and might also underlie the positive correlations of risk and loss aversion in the depressed groups, at least to some degree. Whether the negative correlation of insula activity with loss reflects direct involvement in negative valuation (with lower activity representing loss) or, alternatively, reflects the *regulation* of negative valuation requires further examination in future studies. The insula has been implicated in visceral affects, such as disgust^28^ and pain^29^, as well as aversive outcomes or prediction error^30^. Increased insula activity in a risk-taking decision has also been associated with harm avoidance and neuroticism traits of individuals^31^. Thus, our finding also implicates the insula in instances of heightened negative valuation in depressed patients and perhaps suggests a relationship to heightened negative affect. More specifically, our study has linked abnormal insula activity with both probabilistic loss valuation and suicidal risk.

The suicide attempters also exhibited a disrupted sgACC response to potential gain in the loss aversion task. The disrupted sgACC function in the suicidal patients suggests that a heightened sensitivity to possible negative outcomes could also be related to a lower sensitivity to the possible positive (or neutral) outcomes when they are directly compared. This potential interaction between negative and positive valuation could occur via competition between the valuation processes or via regulatory mechanisms. In fact, the sgACC has been associated with the valuation of reward^12, 32, 33^ and emotion regulation^34^. Abnormally reduced gray matter volume in the sgACC was reported in patients with unipolar and bipolar depression^35^, which appears to be largely due to glia loss^34^. In fact, heightened activity in the region has been associated with depression, with a reduction in activity observed in patients who responded favorably to antidepressant drug treatment^34^. Yet a blunted sgACC response to high expected reward was also identified in a recent fMRI study on depressed elderly suicide attempters performing a reversal learning task^12^. Here we found that the sgACC response to potential gains in the loss aversion task was primarily disrupted in suicide attempters. Its exact role in either valuation or valuation regulation in decisions under uncertainty, especially when gains and losses are directly compared, requires further study. Nonetheless, our finding could represent a partial mechanism for why losses are so acute in suicidal patients: not only do they appear to have heightened sensitivity to possible negative outcomes, it may also be related to a type of contrast effect in which gains are also devalued or otherwise improperly processed when potential gains (or no losses) are compared with potential losses.

The left amygdala response to potential loss in the loss aversion task was blunted in depressed patients regardless of suicide attempt history, which implies a more general relationship of amygdala dysfunction with loss valuation and depression. In previous studies, amygdala lesions have been reported to eliminate loss aversion in decision-making under risk^36^, and gray matter volume in the amygdala has positively correlated with loss aversion across individuals^37^. Behavioral loss aversion and its regulation with a reappraisal strategy were also related to amygdala activity in response to losses relative to gains^22^. Taken together, these findings suggest that normal amygdala function leads to typical loss aversion, a loss of amygdala function (such as from lesions) may lead to an elimination of loss aversion, whereas an active but dysfunctional amygdala may in more severe cases contribute to heightened loss aversion. The specific mechanism by which this would occur could again be due to valuation processing *per se* or regulation of other structures. Regarding emotion regulation’s role in the neural circuitry of decision-making under uncertainty in depressed and suicidal patients, it is noteworthy that loss aversion in the depressed patients positively correlated with the use of an inferior emotion regulation strategy in our study. Thus, an important future direction in the neuroscience of depression and attempted suicide is to delineate the specific roles of the identified brain regions to determine whether the primary source of the heightened negative valuations lies in brain regions mediating valuation processing or those that regulate this process.

### Limitations

We used hypothetical instead of real money reward because of the relatively large payoff amounts and ethical issues in patient studies. However, prior evidence suggests that hypothetical money is a valid proxy for real reward in delay discounting tasks^38^, and previous studies on loss aversion have successfully used hypothetical payoffs^39, 40^. Our results also provide evidence for the validity of hypothetical money, which also speaks to the robustness of the valuation effects. Nevertheless, the effect of magnitude in monetary gain and loss should be carefully assessed, especially for a hypothetical payoff.

The monetary decision-making tasks in the present study are examples of Prospect theory based tasks (decision-making under risk) widely used in neuroeconomics. These are quite stylized types of tasks designed for quantitative estimation of one’s decision preference. However, experimental tasks are usually simplified versions of decision-making in real life (to isolate variables under examination), as real-world decision-making usually involves, e.g.,  incomplete knowledge in probability and/or outcome values, except in certain cases such as lotteries and insurance. Decision-making tasks can continue to strive for more realism in future studies.

Although our tasks examined key factors underlying decision-making, the tasks also require evaluation of numeric values and probabilities. These evaluations may engage cognitive processes such as arithmetic calculation, working memory and those underlying IQ. Therefore, reduced cognitive ability in depression might also be confounded in these tasks. In the present study, we examined age, years of education, and severity of depression symptoms between suicide attempters and non-suicidal patients, and these were not found to be directly correlated with risk or loss aversion. Nonetheless, other factors were not examined, such as IQ scores and other cognitive functions, so we cannot fully exclude the possibility that they may also underlie the observed differences in risk and loss aversion.

In the preliminary fMRI experiment, the number of subjects was limited particularly in the patient subgroups, which yielded a weak sensitivity in the group analysis of the fMRI data. Thus, the fMRI analysis was focused on *a priori* regions of interest using a relatively liberal threshold level with a small volume correction. The fMRI findings in this study are thus provided as a preliminary report only, and further studies with larger groups of patients should be undertaken.

### Conclusion

Heightened risk aversion for potential losses and loss aversion are valuation biases that we uniquely identified in depressed patients with a history of attempting suicide. Risk and loss aversions were also significantly interrelated in the depressed patients, suggesting a common pathophysiological mechanism that is likely related to suicide behavior. The altered valuation function in suicidal depressed patients was related to disrupted neural responses to potential gain and loss in the sgACC, the insula, and the left amygdala, regions involved in valuation, emotion reactivity, and emotion regulation. Our study demonstrated that suicidal depressed patients had uniquely distorted valuations of prospective outcomes even without feedback learning. Both risk and loss aversions reflected unique valuation biases in suicide attempters that could not be captured by conventional clinical questionnaires, and only partially so by emotion regulation assessment; nor was it alleviated by current antidepressant treatment. Thus this neuroeconomics approach is promising as a complementary clinical assessment of potential susceptibility for depression and, in the extreme, potential suicidal risk.

## Methods

### Subjects

We initially recruited 122 patients with unipolar depression (109 outpatients and 13 inpatients; age range 18–44 years), without a history of neurological illness, substance abuse, major head trauma, or seizures. Patients with anxiety disorder were also excluded, but two nonsuicidal patients who participated in the behavioral and fMRI experiments were later diagnosed with anxiety disorder (panic disorder and OCD, respectively). The findings in risk and loss aversion remained unchanged after removing these two participants from the analyses. Diagnostic assessment was conducted by a psychiatrist (JHC) using semi-structured interviews of the Mini-International Neuropsychiatric Interview (M.I.N.I.)^41^. Patients reported moderate to severe symptoms of depression in the Beck Depression Inventory^42^ (BDI: 30.1 ± 10.2, Mean ± S.D.). The depressed patients were divided into 45 previous suicide attempters and 47 without a prior history of any suicide attempts. Suicide attempt history was confirmed with a clinical interview and custom-made questionnaires for a history of suicide attempts and self-harm behaviors; 30 additional depressed patients with inconsistent or insufficient records concerning any prior suicide attempt were excluded. Details in suicide attempt history were varied in 45 suicide attempters. Twenty-one out of 45 patients reported multiple suicide attempts. The interval from the last suicide attempt was shorter than a month in 11 patients, between a month and 12 months for 15 patients, and longer than 12 months in 19 patients. Methods of suicide attempts included overdose (n = 15), cutting (n = 15), hanging (n = 7), vehicles (n = 3), CO2 inhalation (n = 3), leaping (n = 2) and etc.

We recruited 129 healthy participants (age range 19-39 years) from the local community using an advertisement on an internet website. Exclusion criteria for healthy controls were any history of neurological or psychiatric disorder and a history of self-injury or a suicide attempt. Participants with a high score on BDI (>13) and State or Trait Anxiety Inventory (>40) were also excluded from the analysis, yielding 75 healthy participants for the control group. Nevertheless, all findings in this study remained the same as obtained if participants with moderate levels of BDI (up to 16) or STAI (up to 57) were included in the control group. Detailed demographic information of each group is shown in Table 1.

All participants provided written informed consent for the study. All experimental procedures were approved by the Institutional Review Board (IRB) of the Korea Advanced Institute of Science and Technology (KAIST) and the IRB of Seoul St. Mary’s Hospital, The Catholic University of Korea. All experimental procedures followed relevant institutional guidelines and regulations.

For the preliminary fMRI experiment, 22 patients with depressive disorder (10 males; mean age, 27.7 years; range, 18–44 years; 10 suicide attempters) and 22 healthy controls (10 males; mean age, 28.8 years; range, 21–39 years) from the larger behavioral portion of our study participated.

### Behavioral experiment

#### Risk aversion task

The risk aversion task using the probability discounting paradigm^43^ was conducted in both gain and loss conditions, which were alternated across trials. Participants were instructed to choose between a certain and a probabilistic gain in the gain condition trials, and a certain and a probabilistic loss in the loss condition trials (Fig. 1A). The monetary amount for the certain outcome was held constant throughout the experiment at + 20,000 KRW for a certain gain and −20,000 KRW for a certain loss (20,000 KRW is approximately 20 U.S. dollars). In both the gain and loss conditions, 6 different levels of probability (93, 79, 65, 51, 37 or 23%) for the gamble outcome were used across trials (e.g., for the gain condition example in Fig. 1A: P = 51%). The participants in the experiment were instructed to evaluate each option and to decide at their own pace which to choose. In each choice, no feedback was provided for whether the gamble option yielded an actual gain or loss, so that the participants evaluated each option with the explicit knowledge of prospective outcomes and their probability (decision-making under risk with a known probability) without the introduction of reward-based feedback. The locations of both options on the computer screen were counterbalanced to control for any positional bias. The valence condition (gain or loss) and the probability level for the probabilistic outcome were alternated across trials.

The amount of the monetary gain or loss in the gamble option was adjusted dynamically depending on each participant’s choices in the prior two consecutive gambles with the given probability, as in a previous study^43^. For example, when the participant chose a probabilistic gain (gamble choice) with the given probability over a certain gain of $20 in consecutive trials, the monetary gain in the gamble choice was reduced in a stepwise manner. When the participant chose a certain gain of $20 over a probabilistic gain (gamble choice) of the given probability in two successive trials, the monetary gain in the gamble choice was increased. The potential monetary loss in the gamble option in the loss condition was adjusted in a similar manner. The total number of choice trials for the gain and loss condition were approximately 100 each, producing approximately 16 trials per each probability level (16 trials for 6 levels of probability = 96 trials), i.e. about 8 adjustments of the monetary amount in the gamble option. The task was implemented using MATLAB 7.10 (MathWorks, Nattick, MA).

Participants were provided detailed instructions for the task and completed a practice run of 6 trials. At the beginning of the task we included a series of 12 validation trials in which the monetary amount in the certain and probabilistic options were equal; thus, participants should have selected the certain reward over the probabilistic reward (e.g., gain $20 with 100% certainty over $20 with 50% certainty), and the probabilistic punishment over certain punishment (e.g., lose $20 with 50% certainty over $20 with 100% certainty). Participants with poor performance in the validation trials (<6 correct responses out of a possible 12) were considered to lack adequate understanding of the task and were excluded from further analysis (two healthy controls, 4 nonsuicidal patients, and 2 suicide attempters were excluded).

The discount rate *k* was estimated for the gain and loss conditions for each participant by fitting Equation 1 (see Supplementary Fig. 1 for examples of the fitting curve):

1 $${\rm{SV}}=\,\frac{1}{1+k\theta }$$

where, for each probability level, the subjective valuation (SV) is the subjective discount of the probabilistic outcomes (i.e., the calculated indifference point) and *θ* is the odds against winning (probability P):

2 $$\theta =\frac{1-P}{P}\,$$

For example, in the Fig. 1A gain example, P = 0.51, and thus *θ* = 0.49/0.51. For both the gain and loss conditions, k = 1 was risk-neutral. For the gain condition, a value of *k*_*gain*_ > 1 underestimates the probability of the gain outcome and thus increases probability discounting, indicating risk aversion; similarly, *k*_*loss*_ > 1 in the loss condition underestimates the chance of the probabilistic loss, thus preferring probabilistic (risky) loss over a certain loss (note that *k*_*gain*_ and *k*_*loss*_ reflect risk-preference in the opposite directions). Because the discount rates *k*_*gain*_ and *k*_*loss*_ across participants exhibited right-skewed distributions, we took the logarithmic value of *k* to normalize their distributions and defined “log *k*_*gain*_” and “ −log *k*_*loss*_” as our measures of risk aversion in the gain and loss conditions, respectively (see Supplementary Fig. 2 for distribution of the discount rates *k*_*gain*_ and *k*_*loss*_ and their logarithm values). Thus, logarithmic values for both gains and losses that were greater than 0 indicated a risk-averse propensity, and values lower than 0 indicated a risk-taking propensity. The log-transformed k-values were used in the ANOVAs for group comparisons of risk aversion.

#### Loss aversion task

In the loss aversion task, the participants repeatedly decided whether to take a 50-50 gamble or not, as shown in Fig. 1B. The amount of loss in the gamble was fixed at two low and high stakes conditions: 20,000 and 100,000 KRW, which were approximately $20 and $100 in U.S. dollars. The amount of gain in the gamble was then dynamically adjusted for each stakes level using a previously described algorithm^38^. Briefly, the outer limits of the ratio of gain to loss at the beginning of the task were set to [0, 8], in which a ratio of 0 meant a gain amount of nothing, and a ratio of 8 meant a gain amount 8 times more than the loss amount. As a subject made successive choices, both outer limits for each stakes condition approached the indifference point at which the subjective value of the gamble equaled $0, the status quo. Again, there was no feedback for whether the 50-50 gamble option yielded an actual gain or loss, in order to exclude any confounding effect of feedback learning. The algorithm was terminated at the indifference point, where the difference between the two outer limits was less than 2,000 KRW. Loss aversion *λ* was then calculated as the ratio of the amount of gain to the amount of loss at the indifference point, such that *x* units of gain equals *y* units of loss, with *x* greater than *y* for loss aversion^38^. We estimated loss aversion *λ* for low and high stakes levels respectively, then used the average of these two *λ* values in the analysis.

### Psychometric questionnaires

Participants were asked to complete the following psychometric questionnaires a week after the behavioral experiment: the Beck Depression Inventory^42^, the Beck Hopelessness Scale^44^, the Beck Scale for Suicide Ideation^45^, the State and Trait Anxiety Inventory^46^, the Barratt Impulsiveness Scale^47^, the Behavioral Inhibition and Activation Scales (BIS/BAS)^48^, and the Emotional Regulation Questionnaire (ERQ)^20^. The questionnaires collected from the depressed patients were limited in number (47 of 92) because of technical issues, such as having no subsequent follow-up visits.

### Statistical analysis

The statistical analyses were conducted using SPSS 17.0 software (SPSS Inc., Chicago, IL). The behavioral measures from the decision-making tasks and the questionnaire scores were compared across groups using a one-way ANOVA (Tukey’s test as the post hoc analysis). Pearson’s correlation R and p-value were also estimated across age, years of education, psychometric questionnaire scores and the behavioral measures (i.e. risk and loss aversions) for the depressed patients. Fisher’s z transformation was used to compare the Pearson’ correlation R observed in the depressed patients vs. healthy controls.

### Preliminary fMRI experiment

#### Experimental tasks

The probability discounting task conducted during the fMRI scan consisted of 144 trials (72 gain and 72 loss conditions). In each trial, both options were presented for 6 seconds, as shown in Fig. 1, and the participants were instructed to make their decision using a button press. A fixation mark was displayed with a varying duration of 2 to 5 seconds after the decision phase. The probabilistic monetary gain and loss for each participant were adjusted close to his or her indifference point, which was estimated from the preceding behavioral experiment, to ensure that the participant would select both probabilistic and certain options during the fMRI task.

In the fMRI loss aversion task, the participants were asked to choose between a status quo or the 50-50 gamble as shown in Fig. 1. The subjective value of the 50-50 gamble can be estimated with loss aversion λ, the subjective weight of loss over gain, which was assessed in the loss aversion task in the behavioral experiment. The monetary loss in the 50-50 gamble varied from 80,000 to 120,000 KRW, and the monetary gain varied from 0 to 960,000 KRW. The ratio between monetary gain and loss (potential loss aversion) was set to a range of 0–1 (25 trials), 1–2 (25 trials), 2–4 (25 trials) and 4–8 (25 trials) to ensure that every participant had both accepted and rejected gambles. Each gamble was shown for 3 seconds, followed by a fixation cross with a variable duration of 2–4 seconds.

#### fMRI data acquisition

The imaging data were collected using a 3.0 T Siemens Verio scanner (Siemens, Munich, Germany) with a standard quadrature head coil. The participant’s head was immobilized using foam pads to minimize motion artifacts. The task stimuli were projected on a LCD screen behind the MRI scanner, and the screen was visible for the subject through a mirror mounted above the subject’s head. A Lumina response pad for fMRI (Cedrus co., CA) with two buttons was used for the subject’s responses. E-prime 1.1 software (Psychology Software Tools Inc., PA), which was run on a PC, was utilized to control the display of the task stimuli and to record the subject’s responses during the fMRI session.

The functional images covered the entire brain and were acquired using a gradient echo-planar image (EPI) pulse sequence with the following parameters: field of view = 192 mm, matrix size = 96 × 96, number of slices = 32, slice thickness = 4.0 mm, in-plane resolution 2 × 2 mm, TE = 27 ms, TR = 2000 ms, and flip angle = 90°. The acquisition planes were tilted approximately 30° from the anterior-posterior commissure line to minimize artifacts that resulted from the air-tissue interface in the orbitofrontal region. The first 3 volumes in each run were automatically discarded to enable the EPI signal to stabilize. After the EPI scans for probability discounting and loss aversion (694 and 310 volumes, respectively), a T1-weighted anatomical scan was collected using an MP-RAGE pulse sequence with field of view 240 mm, matrix size = 256 × 256, number of slices = 192, slice thickness = 0.94 mm, in-plane resolution = 0.94 × 0.94 mm, TE = 2.46 ms, TR = 1900 ms, and flip angle = 9°.

#### Image processing

Image processing was conducted using SPM8 (http://www.fil.ion.ucl.ac.uk/spm). After slice timing correction, images were realigned to the first volume in each run to minimize potential artifacts from head movement. The mean image was coregistered with the T1 anatomical images, and the images were normalized to a standard stereotactic space (Montreal Neurological Institute template) using a transformation parameter derived from the normalization of the T1 anatomical image. The normalized images were then spatially smoothed to minimize noise using a Gaussian filter of 8 mm full-width at half-maximum.

The blood-oxygen-level-dependent (BOLD) signal was analyzed using a general linear model (GLM), in which each event was modeled using the canonical hemodynamic response function. In the probability discounting task, trials were divided into gain and loss trials according to the type of outcome. The regressors of interest in the GLM were the subjective value estimated with risk aversion in each individual, the magnitude of monetary gain or loss, and the odds against win or loss probability in the probabilistic choice of each trial (3). In the GLM for the loss aversion task, BOLD activity at choice phase was modeled by the amounts of monetary gain and loss in the 50-50 gamble of each trial to identify the brain activity that correlated with the potential gain and loss (11). Head movement parameters were also included in both GLMs as regressors of no interest. The beta estimates (the level of BOLD covariation with above experimental variables) computed from each individual were entered into a second level (random effects) group comparison using a one-way ANOVA, which revealed significant differences in BOLD activity across the previous suicide attempters, nonsuicidal depressed patients, and healthy controls. A significance level of uncorrected p < 0.005 (with an extent threshold of k > 40) and small-volume correction were applied for regions of *a priori* interest: the striatum, orbitofrontal cortex, ventromedial prefrontal cortex, ventral anterior cingulate cortex and midbrain dopaminergic region, as reported in a previous study (11), as well as the amygdala and insula regions related to negative affect. As a post-hoc analysis, beta contrasts were averaged in each ROI of a significant group difference and significance levels were compared using Tukey’s test. For exploratory purposes, a whole-brain analysis of between- and within-group effects were tested with a significance level of uncorrected p < 0.001 and an extent threshold k > 10.

## Electronic supplementary material

Supplementary Information
