# Supplementary material for: Heightened aversion to risk and loss in depressed patients with a suicide attempt history
Source: Sci Rep. 2017 Sep 11;7:11228. doi: 10.1038/s41598-017-10541-5 (PMC5593974; doi:10.1038/s41598-017-10541-5)

Supplementary Information

Heightened aversion to risk and loss in depressed patients with a suicide attempt history

Kwangyeol Baek[1,2,§], Jae-Hyung Kwon[1,§], Jeong-Ho Chae[3,*], Yong An Chung[4], Jerald D. Kralik[1], Jung-Ah Min[3], HyuJung Huh[3], Kyung Mook Choi[3], Kuk-In Jang[3], Na-Bin Lee[3], Sunyoung Kim[3], Bradley S. Peterson[5], and Jaeseung Jeong[1,*]

[1] Department of Bio and Brain Engineering, Korea Advanced Institute of Science and Technology (KAIST), Daejeon, 305-701, Republic of Korea

[2] Department of Psychiatry, University of Cambridge, Addenbrooke's Hospital, Cambridge CB2 0QQ, United Kingdom

[3] Department of Psychiatry, College of Medicine, The Catholic University of Korea Seoul, 137-701, Republic of Korea

[4] Department of Radiology, College of Medicine, The Catholic University of Korea Seoul, 137-701, Republic of Korea

[5] Institute for the Developing Mind, Children's Hospital Los Angeles and the Keck School of Medicine at the University of Southern California, 90033 United States

[§]: K.B and J. K contributed equally to this study

Table S1. Group comparison of risk and loss aversion within depressed patients with respect to current suicide ideation (A) and antidepressant treatment (B).

A. Current suicide ideation

Behavioral measures, Mean (SD)	Suicide ideation (N = 49)	No ideation (N = 43)	*p* value[a]
Risk aversion			
Gain condition	0.33 (0.29)	0.35 (0.28)	0.53
Loss condition	-0.03 (0.37)	0.03 (0.31)	0.95
Loss aversion	2.71 (2.10)	2.72 (2.07)	0.97

[a] Two-sample t-test.

B. Current antidepressant treatment

Behavioral measures, Mean (SD)	Medication (N = 50)	No medication (N = 42)	*p* value[a]
Risk aversion			
Gain condition	0.33 (0.34)	0.35 (0.27)	0.83
Loss condition	-0.05 (0.36)	0.07 (0.30)	0.10
Loss aversion	2.78 (2.19)	2.64 (1.95)	0.74

[a] Two-sample t-test.

Table S2. BOLD activity significantly correlated with the subjective value of probabilistic gain within each group (Risk aversion task). $p < 0.001$, uncorrected.

Location	*x*	*y*	*z*	*Cluster extent*	t-statistics
**Healthy controls**					
R putamen [a]	20	2	0	14	5.43
**Nonsuicidal depressed patients**					
L superior temporal gyrus [b]	-50	4	-10	70	-10.57
**Suicide attempters**					
R amygdala / uncus [b]	30	4	-24	19	-11.48
R insula [b]	42	-2	-8	164	-10.45
L middle temporal gyrus [b]	-66	-12	-16	13	-10.24
L temporal pole [b]	-52	14	-26	26	-8.85
L insula [b]	-46	-18	6	37	-7.73
L globus pallidus [b]	-18	-10	-6	23	-7.47
R inferior frontal gyrus [b]	24	22	-18	18	-5.50

[a]: Positive correlation

[b]: Negative correlation.

Table S3. BOLD activity negatively correlated with the subjective value of probabilistic loss within each group (Risk aversion task). $p < 0.001$, uncorrected. No positive correlation was found.

Location	***x***	***y***	***z***	***Cluster extent***	**t-statistics**
**Healthy controls**					
R parahippocampal gyrus / cerebellum	22	-28	-26	79	-6.87
Midbrain	0	-12	-8	23	-4.82
L thalamus	-16	-10	-4	15	-4.63
**Suicide attempters**					
L insula	-44	-8	18	28	-8.68

Table S4. BOLD activity that significantly covariated with the amount of gain in 50-50 gambles within each group (Loss aversion task). $p < 0.001$, uncorrected. All positive correlations.

Location	***x***	***y***	***z***	***Cluster extent***	**t-statistics**
**Healthy controls**					
L superior temporal gyrus / parahippocampal gyrus	-36	2	-20	79	6.82
R mOFC	2	32	-22	88	6.02
R vmPFC	8	46	-12	59	4.44
R subgenual ACC	2	26	-6	70	5.61
L subgenual ACC	-6	52	-4	36	4.55
L PCC/Precuneus	-14	-62	16	91	5.22
L cerebellum	-18	-34	-22	29	5.01
L parahippocampal gyrus	-32	-28	-20	33	4.92
L middle temporal gyrus	-52	-6	-20	28	4.78
**Nonsuicidal depressed patients**					
L Subgenual ACC / ventral striatum	-8	16	-12	20	6.71
L middle temporal gyrus	-52	-12	-20	20	5.70
**Suicide attempters**					
R precentral / postcentral gyrus	40	-24	62	342	7.13
L supplementary motor area	-12	-12	58	22	6.41
L precentral gyrus	-46	-4	48	41	5.62

Table S5. BOLD activity that significantly covariated with the amount of loss in 50-50 gambles within each group (Loss aversion task). $p < 0.001$, uncorrected. Positive correlation.

Location	***x***	***y***	***z***	***Cluster extent***	**t-statistics**
**Healthy controls**					
L inferior frontal gyrus	-44	32	-4	14	3.96

SI Figures

Figure S1. Examples of fitting of the discount rate k with the subjective discount values in probabilistic outcome. Here are shown the indifference point values across probability levels and the fitting curves from two example data (Blue: k = 0.56, Red: k = 7.05).

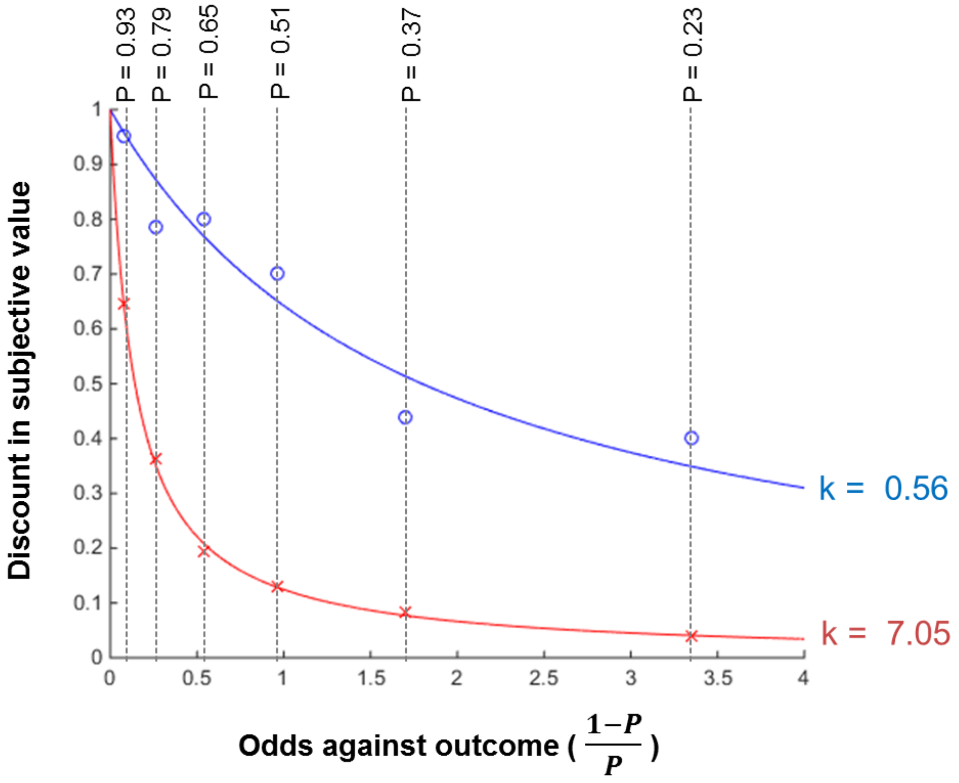

Figure S2. Distribution of the original discount rates k and their logarithm values across subjects. (A) Original discount rates k exhibited right-skewed distribution. (B) Logarithm values of discount rates k (log k) were closer to the normal distribution.

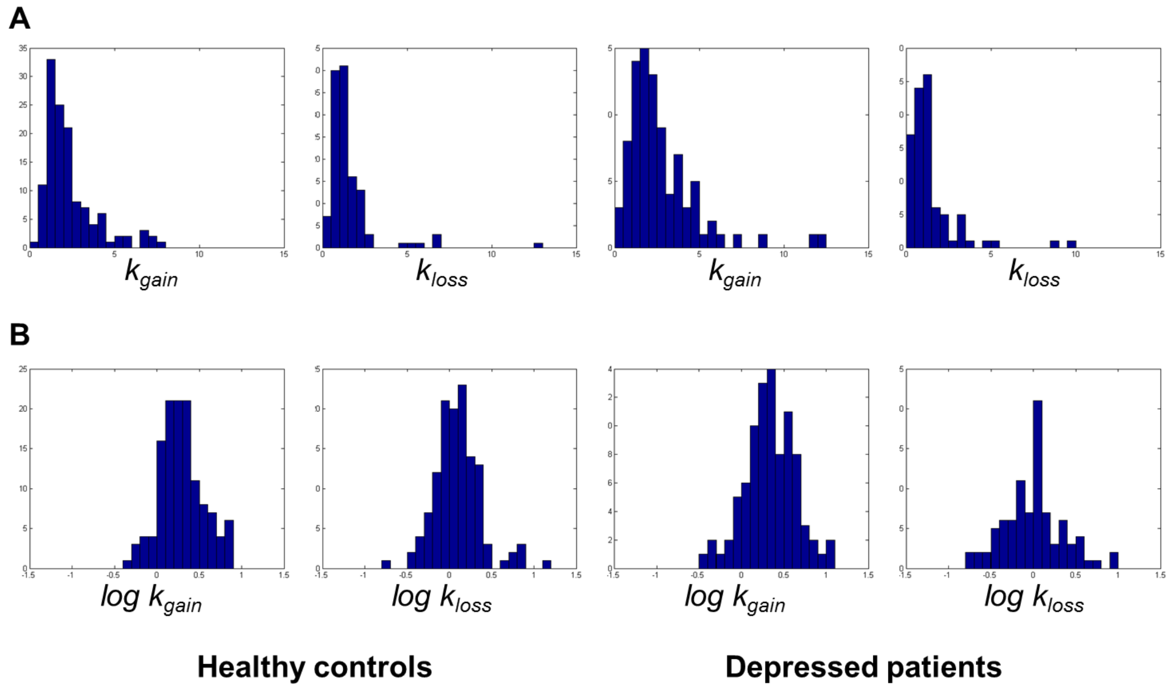

Supplement: Supplementary file 1 — Supplementary Information [file 41598_2017_10541_MOESM1_ESM.pdf]
